# Thermoelectric Properties of Highly-Crystallized Ge-Te-Se Glasses Doped with Cu/Bi

**DOI:** 10.3390/ma10040328

**Published:** 2017-03-23

**Authors:** Bhuvanesh Srinivasan, Catherine Boussard-Pledel, Vincent Dorcet, Manisha Samanta, Kanishka Biswas, Robin Lefèvre, Franck Gascoin, François Cheviré, Sylvain Tricot, Michael Reece, Bruno Bureau

**Affiliations:** 1 Équipe Verres et Céramiques, ISCR CNRS UMR 6226, Université de Rennes 1, Rennes 35042, France; bhuvanesh.srinivasan@univ-rennes1.fr (B.S.); catherine.boussard@univ-rennes1.fr (C.B.-P.); francois.chevire@univ-rennes1.fr (F.C.); 2 PRATS, ISCR CNRS UMR 6226, Université de Rennes 1, Rennes 35042, France; vincent.dorcet@univ-rennes1.fr; 3 New Chemistry Unit, Jawaharlal Nehru Centre for Advanced Scientific Research, Bangalore 560064, India; manishas@jncasr.ac.in (M.S.); kanishka@jncasr.ac.in (K.B.); 4 ENSICAEN, UNICAEN, CNRS, IUT-Caen, CRISMAT, Normandie Université, Caen 14050, France; robin.lefevre@ensicaen.fr (R.L.); franck.gascoin@ensicaen.fr (F.G.); 5 Institut de Physique de Rennes, CNRS UMR 6251-Université de Rennes 1, Rennes 35042, France; sylvain.tricot@univ-rennes1.fr; 6 School of Engineering and Materials Science, Queen Mary University of London, London E1 4NS, UK; m.j.reece@qmul.ac.uk

**Keywords:** chalcogenide glasses, heavy doping, complete crystallization, thermal conductivity, power factor, thermoelectrics

## Abstract

Chalcogenide semiconducting systems are of growing interest for mid-temperature range (~500 K) thermoelectric applications. In this work, Ge_20_Te_77_Se_3_ glasses were intentionally crystallized by doping with Cu and Bi. These effectively-crystallized materials of composition (Ge_20_Te_77_Se_3_)_100−*x*_M*_x_* (M = Cu or Bi; *x* = 5, 10, 15), obtained by vacuum-melting and quenching techniques, were found to have multiple crystalline phases and exhibit increased electrical conductivity due to excess hole concentration. These materials also have ultra-low thermal conductivity, especially the heavily-doped (Ge_20_Te_77_Se_3_)_100−*x*_Bi*_x_* (*x* = 10, 15) samples, which possess lattice thermal conductivity of ~0.7 Wm^−1^ K^−1^ at 525 K due to the assumable formation of nano-precipitates rich in Bi, which are effective phonon scatterers. Owing to their high metallic behavior, Cu-doped samples did not manifest as low thermal conductivity as Bi-doped samples. The exceptionally low thermal conductivity of the Bi-doped materials did not, alone, significantly enhance the thermoelectric figure of merit, zT. The attempt to improve the thermoelectric properties by crystallizing the chalcogenide glass compositions by excess doping did not yield power factors comparable with the state of the art thermoelectric materials, as these highly electrically conductive crystallized materials could not retain the characteristic high Seebeck coefficient values of semiconducting telluride glasses.

## 1. Introduction

Coupled with the severe exploitation of fossil fuels and an ever-increasing demand for a sustainable supply of energy, the hunt for high-performance thermoelectric materials has gained greater momentum over the past decade due to their ability to directly convert thermal and electrical energy and provide an alternative route for power generation and refrigeration [1,2,3]. Efficient thermoelectric devices have great potential to convert waste heat from power plants, automotive engines, and industrial processes into fruitful electricity.

A thermoelectric material’s potential to convert waste heat into electricity is quantified by a dimensionless figure of merit, *zT*, as given by Equation (1):

(1) $$zT=\frac{S^{2}\sigma T}{\kappa_{total}}$$

where *S*, σ, *T*, and $\kappa_{total}$ are the Seebeck coefficient, electrical conductivity, temperature, and total thermal conductivity, respectively. Ideal thermoelectric materials are based on the “phonon glass electron crystal” (PGEC) model [4,5], which means that the materials must concurrently possess low lattice thermal conductivity, as in the case of a glass where phonons are effectively scattered, as well as have high electrical conductivity, like a crystal where there is a high level of electron mobility. The fact that these thermoelectric transport properties are highly interrelated creates a greater challenge in enhancing *zT*. Advances in recent times shows that it is feasible to enhance *zT* by a number of approaches: quantum confinement of electron charge carriers [6]; synergistic nano-structuring [7,8,9,10]; nano-inclusions, which enable acoustic phonon scatterings [11,12]; electron filtering [13]; convergence of electronic band valleys [14,15,16]; fostering resonant levels by impurities inside the valence band [17]; alloying to create point defects [18]; and complex crystal structures, like skutterudites [19,20], Zintl compounds [21,22], and hetero-structured superlattice thin-films [23].

In the past few years, the idea of thermoelectric glasses has gained some limelight. Telluride glasses, particularly known for their low thermal conductivity of 0.12 WK^−1^ m^−1^ [24] and simple glass-making process, makes them ideal candidates. An array of compositions of chalcogenide semiconducting glasses and glass-ceramics with low thermal conductivity and unusually high electrical conductivity for a glassy phase have been previously reported [25,26,27]. Though these kind of semiconducting glasses, especially Cu-doped telluride glasses, exhibit high Seebeck coefficient of around 600 µV/K at room temperatures [25,26,28,29,30,31,32], their high degree of structural disorder causes large electron scatterings that results in low mobility and electrical conductivity, which pulls down the power factor and overall *zT* to values that are too low for any relevant large-scale industrial applications.

Though PbTe, an extensively-studied chalcogenide, has proved its mettle in thermoelectric efficiency, the toxic nature of Pb limits their practical applications. The other budding prospect from the semiconducting IV–VI group is the GeTe based alloys. Carrier and phonon engineering of solid-state solutions of GeTe, partially substituted with one/more of these elements like Pb, Sb, Ag, Co, Mn, and Yb has shown promising *zT* > 1 in the intermediate temperature ranges [33].

Previous reports [34,35] on Te-rich, high-purity Ge_20_Te_77_Se_3_ ternary glasses focused on optical fibers and far infra-red sensing applications due to their good transparency to long wavelengths and inherently low level of optical losses. These GeTe-based stable glass systems, though extensively studied for optical purposes, are not well explored for thermoelectric applications, especially the crystallized composition of these glasses.

Understanding the fact that pure glass phase alone will not produce materials with high *zT* values, in this study, we employed a different approach of intentional crystallization of Ge–Te–Se glass compositions by heavily doping with Cu and Bi, which collapses the glassy network.

Herein, we report an ultra-low lattice thermal conductivity of ~0.7 Wm^−1^ K^−1^ at 525 K along with increased electrical conductivity due to excess charge carrier concentration and the unfavorably reduced Seebeck co-efficient values in p-type, high-quality, multi-phased crystalline ingots of composition (Ge_20_Te_77_Se_3_)_100−*x*_Bi*_x_* (*x* = 10, 15).

## 2. Materials and Methods

### 2.1. Reagents

Ge (Umicore, Olen, Belgium, 5N), Te (JGI, Brussels, Belgium, 5N), Se (Umicore, Olen, Belgium, 5N), Cu (Alfa Aesar, Karlsruhe, Germany, 99.999%), Bi (Strem Chemicals, Newburyport, MA, USA, 99.999%) were used for synthesis without involving any further purification processes.

### 2.2. Synthesis

The samples of (Ge_20_Te_77_Se_3_)_100−*x*_M*_x_* (M = Cu or Bi; *x* = 0, 5, 10, 15) were synthesized using the conventional melt quenching method. Appropriate stoichiometric amounts of the starting elements of Ge, Te, Se, Cu, or Bi were introduced in a fused silica tube (diameter ~10 mm) that had previously been cleaned with hydrofluoric (HF) acid and distilled water and dried under vacuum. The ampoules were sealed under a vacuum of 10^−6^ Torr, then placed in a rocking furnace and slowly heated up to 850 °C over a period of 8 h, then held at that temperature for 12 h before being quenched in water. The tubes were then annealed at 100 °C for 3 h. The obtained ingots were cut and polished to required shapes and dimensions for various thermoelectric measurements.

### 2.3. Powder X-ray Diffraction

X-ray diffraction (XRD) patterns were recorded at room temperature in the 2θ range 15°–90° with a step size of 0.026° and a scan time per step of 400 s using a PANalytical X’Pert Pro diffractometer (PANalytical, Almelo, The Netherlands, Cu-Kα radiation, λ = 1.5418 Å, PIXcel 1D detector). Data Collector and HighScore Plus software packages were used, respectively, for recording and analyzing the patterns. The Rietveld refinement for quantitative analysis was carried out with the Fullprof program [36].

### 2.4. Hall Measurement

The Hall measurements were carried out at room temperature using a home-made four-point probe setup (Van der Pauw method), where a fixed magnetic field of 0.112 T and DC current of 15 mA was applied. The measurements were done on a square shaped sample of dimension ~5 × 5 × 2 mm^3^. The carrier concentration (*n*) and mobility (µ) were computed from carrier sheet density (*n_s_*), sheet resistance (*R_s_*), and Hall Voltage (*V_H_*) using the following equations:

(2) ns=nt=IBe|VH|

(3) µ=1(ensRs)

where *e*, *B*, *I*, and *t* are the charge of the electron, magnetic field, current, and thickness of the sample respectively.

Values of carrier density obtained were robust with an error of less than 2%.

### 2.5. Electrical and Thermal Transport

The electrical conductivity and Seebeck coefficients were measured simultaneously from room temperature to 523 K using a commercial ZEM-3 instrument (ULVAC Co. Ltd., Kanagawa, Japan), under partial pressure of helium. The measurements were made on parallelepiped-shaped samples of dimensions ~10 × 2 × 2 mm^3^.

Thermal diffusivity, *D*, was directly measured from room temperature to 523 K using the laser flash diffusivity method (LFA 457, Netzch Co. Ltd., Selb, Germany). Disc-shaped samples of 10 mm diameter and ~2 mm thickness were used for the measurements. The temperature-dependent heat capacity, *C_p_*, was derived using a standard sample (pyroceram) in LFA-457, which is in good agreement with the Dulong–Petit *C_p_* value. The total thermal conductivity, $\kappa_{total}$ was calculated using the Equation (4):

(4) $$\kappa_{total}=DC_{p}\rho$$

where ρ is the density of the sample. The density of the discs were measured using Archimedes’ principle.

The uncertainty for the measurement of electrical conductivity is ~3%, Seebeck coefficient is ~5%, and thermal conductivity is ~7%.

### 2.6. Microscopic Analysis

Scanning electron microscopy (SEM) and energy dispersive X-ray spectroscopy (EDX, Oxford Instruments, Oxfordshire, UK) analysis were performed using a JEOL JSM 7100F microscope (JEOL, Tokyo, Japan) on polished bulk surface of the samples. Transmission electron microscopy (TEM) investigations were carried out (HRTEM, JEOL 2100F, JEOL) on electron-transparent samples that were prepared by polishing, dimpling, and ion beam milling.

## 3. Results and Discussion

The batch of samples of compositions (Ge_20_Te_77_Se_3_)_100−*x*_M*_x_* (M = Cu or Bi; *x* = 0, 5, 10, 15) that were prepared by vacuum sealed-tube melt quenching technique are denoted as in Table 1.

The samples GTS and GTS-Cu05 were found to be stable glasses (∆T ~ 100 K), while GTS-Cu10 was found to be a partially crystallized glass. The electrical conductivities of these samples were extremely low, so they are not presented in this article (please refer to Supplementary Information, Figures S1 and S2 for information regarding these glassy samples). It is worth noting that the glassy network in GTS is being completely destroyed with addition of more than 10 at % Cu and 5 at % of Bi. The paper focusses only on the thermoelectric-related properties of the completely-crystallized compositions of GTS-Cu15, GTS-Bi05, GTS-Bi10, and GTS-Bi15.

**Figure 1:**
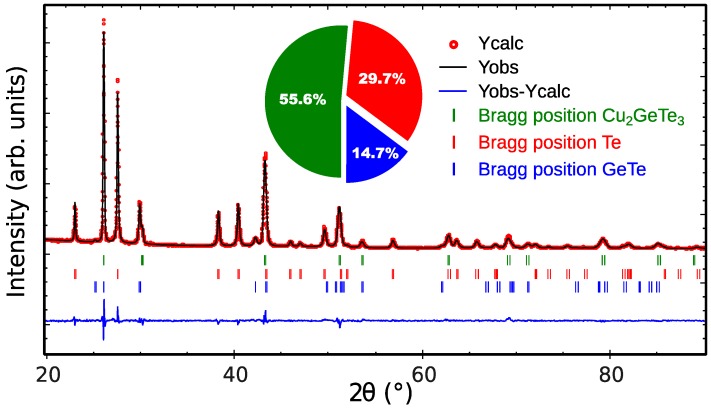
Rietveld refinement pattern for the GTS-Cu15 sample-observed (black line), calculated (red), and difference (blue line) XRD diffraction profiles. The vertical markers correspond to the position of the Bragg reflections for the different phases. The inset pie-chart illustrates the weight contribution of the different phases in the sample.

Powder X-ray diffraction (PXRD) results for GTS-Cu15 (Figure 1) show that the samples were well crystallized and three major phases exists, namely Cu_2_GeTe_3_, Te and GeTe. The peaks for Cu_2_GeTe_3_ phase were indexed based on a cubic blende-type structure with *F*$\bar{4}$3*m* space group (n°216) [37], considering Cu and Ge atoms sharing the same lattice position without any cation ordering, while the Te and GeTe peaks were indexed based on the trigonal structure with the *P*3_1_21 space group (n°152) and rhombohedral structure with *R*3*m* (n°160) space group, respectively. The weight ratios of these phases indicated in the inset pie-chart of Figure 1 shows that Cu_2_GeTe_3_ as the main phase in GTS-Cu15 sample. Refinement details are summarized in supplementary material (Supplementary Information, Table S1).

**Figure 2:**
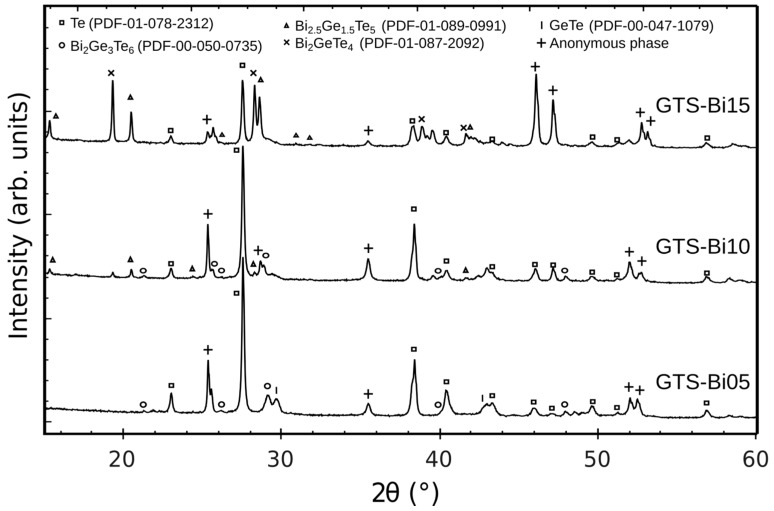
PXRD patterns for Bi-doped GTS samples showing peaks arising from multiple crystalline phases.

PXRD analyses performed on Bi-doped GTS samples are represented in Figure 2, show that all samples contain a bulk proportion of crystalline Te phase (PDF#078-2312, space group *P*3_1_21, n°152) and various Bi-containing phases depending on the initial experimental composition. For instance, GTS-Bi05 contains Bi_2_Ge_3_Te_6_ phase (PDF#050-0735, space group *R*3*m*, n°160), GTS-Bi10 contains small amounts of Bi_2_Ge_3_Te_6_ and Bi_2.5_Ge_1.5_Te_5_ (PDF#089-0991, space group $P\bar{3}$*m*1, n°164) phases, while increasing the Bi content favors the crystallization of Bi-rich Bi–Ge–Te phases, as GTS-Bi15 exhibits a much larger contents of Bi_2.5_Ge_1.5_Te_5_ and Bi_2_GeTe_4_ (PDF#087-2092, space group *R*3*m*, n°166).

At this point, it is essential to mention that in Bi-doped samples an anonymous phase that could not be indexed based on the current available crystallographic databases is present in considerable proportion. This could be a new phase of Bi–Ge–Te and the exact composition of this phase was difficult to estimate in SEM-EDX.

**Figure 3:**
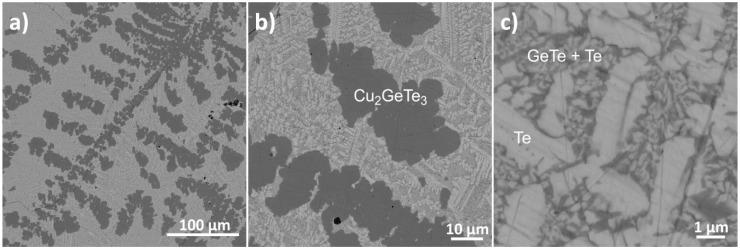
SEM images of GTS-Cu15 sample, (**a**,**b**) shows two distinct regions, the dark grey region is identified as the Cu_2_GeTe_3_ major phase (dendritic growth) and a brighter region; and (**c**) the higher magnification image of bright region was found to be a mixture of Te and GeTe phases.

The SEM images of GTS-Cu15 with different levels of magnification (Figure 3) show several dark patchy domains (dendritic formation) in the backdrop of brighter regions. EDX analysis found that the dark domains correspond to the main phase of Cu_2_GeTe_3_ while the bright matrix appear to be predominantly Te and GeTe phases, establishing solid agreement with XRD and refinement results. Figure 3a clearly shows that Cu_2_GeTe_3_ grows as dendrites. In short, Te-rich phases comprising GeTe in minor proportions are embedded in the Cu_2_GeTe_3_ main phase.

**Figure 4:**
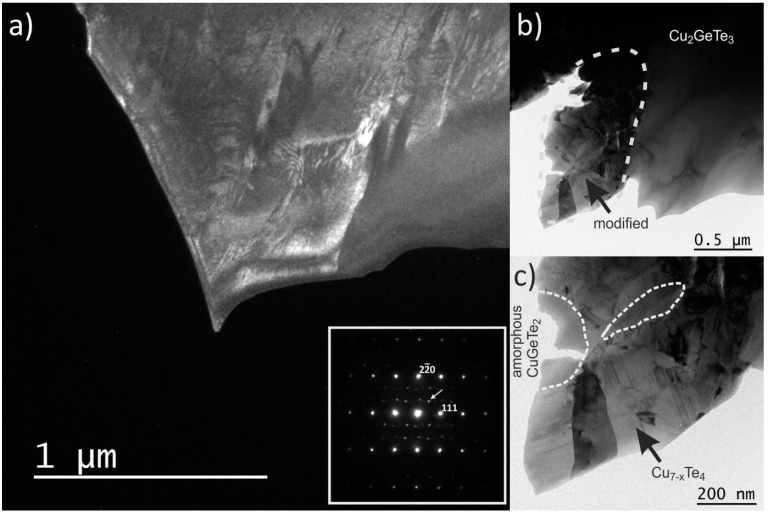
TEM micrographs of GTS-Cu15 (**a**) Low magnification dark field (DF) image of a Cu_2_GeTe_3_ crystallite domain. Inset shows the SAED pattern of the left part of the crystal showing strong reflections that can be indexed in the cubic cell of Cu_2_GeTe_3_. The DF image is made with one of these reflections (see arrow); (**b**) Bright field (BF) image of a Cu_2_GeTe_3_ crystallite phase. The dotted region on the BF image is the one that was apparently modified by ion beam milling; (**c**) Enlargement of the modified area showing the segregation between crystalline Cu_7-*x*_Te_4_ and an amorphous phase of composition CuGeTe_2_.

Furthermore, TEM micrographs on these GTS-Cu15 samples, as in Figure 4, show large and well-dispersed crystallized regions of the Cu_2_GeTe_3_ phase. The Figure 4a presents a dark field (DF) image obtained from the reflection pointed at by an arrow in the inset. The inset presents the selected area electron diffraction (SAED) pattern of the crystal where the strong reflection are indexed in the cubic cell of Cu_2_GeTe_3_ and the weak ones could not be indexed. The left part of this crystal phase shows a contrast between white and grey areas, whereas the right part shows a homogeneous grey contrast. Figure 4b shows a more magnified image of a Cu_2_GeTe_3_ crystallite zone in bright field (BF), where a large and homogeneous crystal of Cu_2_GeTe_3_ main phase and a small polycrystalline area (dotted region) are observed. EDX analysis on this dotted region found it to have, on average, the same composition as that of the main phase. Enlargement of this modified surface, Figure 4c, shows a mixture of crystallized and amorphous regions which were found to be Cu_7−*x*_Te_4_ and CuGeTe_2_ phases respectively by electron diffraction and EDX. As these phases were unidentified in PXRD and SEM, they could have evolved during the sample preparation process of ion beam milling; some regions of the main Cu_2_GeTe_3_ phase that were close to the ion milled area were dissociated into crystalline Cu_7−*x*_Te_4_ and amorphous CuGeTe_2_ phases. The presence of stacking faults in the Cu_7−*x*_Te_4_ phase of the modified region are explained pictorially using HRTEM images in the Supplemental Information (Figure S4). Interesting features that could kindle the thermoelectric properties like nanostructured defect layers or mesostructured grain boundaries were non-existent for this heavily Cu-doped GTS-Cu15 sample.

**Figure 5:**
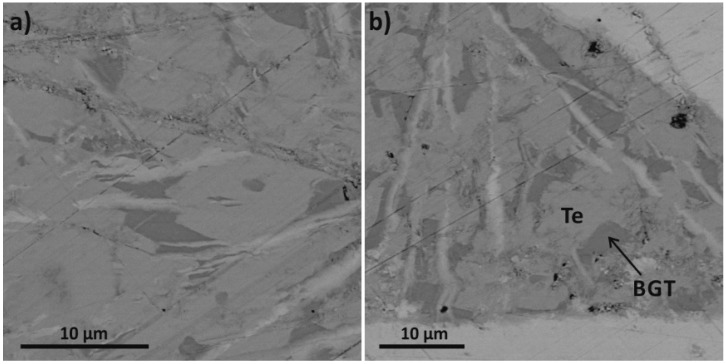
(**a**,**b**) SEM images of GTS-Bi15 sample showing regions of Te phase (pale bright) and Bi-Ge-Te (BGT) phase (darker regions).

Figure 5 displays SEM images of GTS-Bi15 where two major phases are visible, a pale bright region and another darker region. EDX analyses found the bright region to be Te phase and the grey region to be Bi-Ge-Te phase (BGT) with variable compositions, especially the Bi/Ge ratio. This tentatively matches with the PXRD results as well.

**Figure 6:**
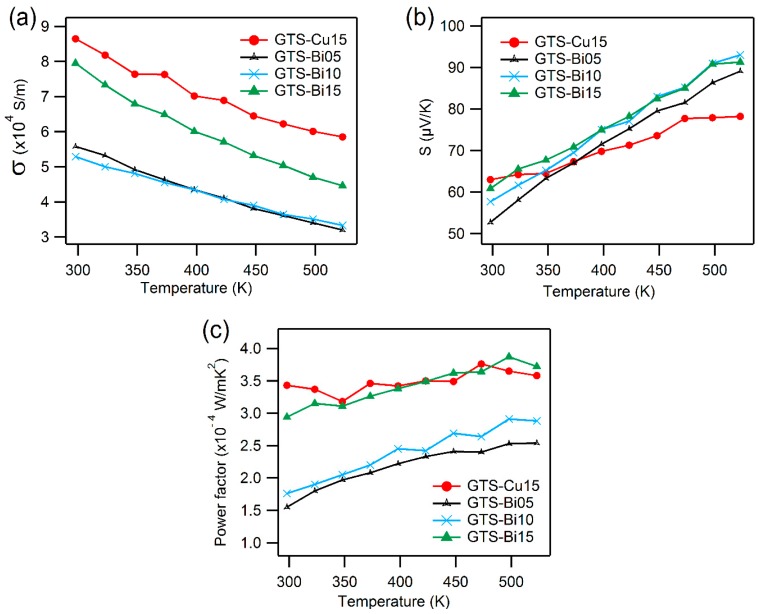
Electrical transport properties: (**a**) Electrical conductivity, σ; (**b**) Seebeck coefficient, S; and (**c**) thermoelectric power factor (S^2^σ), as a function of temperature.

The electrical conductivity as a function of temperature of the GTS samples is presented in Figure 6a. With increasing temperature, the electrical conductivity of all of the samples decreases, which is the archetypal behavior of a degenerate semiconductor [38,39]. Since the Hall voltage is positive in all these samples, holes are the major charge carriers (*p*-type). Results from Hall measurements tabulating the carrier concentration (*n*) and mobility (*µ*), which were calculated using Equations (2) and (3), are presented in Table 2. With an increase in Cu/Bi content, the electrical conductivity increases due to coherent raising of the carrier concentration values and the transformation from a glassy state to a completely crystallized form (i.e., transition from a glassy state of GTS to crystalline GTS-Cu10 or GTS-Bi05 and further).

It is interesting to note the variation of mobility in Bi-doped samples. Despite GTS-Bi10 having twice the carrier concentration values of GTS-Bi05, its carrier charge mobility is reduced by half and this cumulative effect is observed in Figure 6a, where the electrical conductivities of both of these samples are almost the same. It seems like there is a threshold for the increase in conductivity versus Bi content. GTS-Bi15 exhibits much higher conductivity due to high charge carrier density and mobility. It is also seen that Cu doped GTS samples are more electrically conductive compared to the Bi-doped ones, due to high carrier concentration and hole mobility. It is apparent that excess doping of Cu/Bi creates additional vacancies in the GTS network, which is reflected in the enhancement of charge carrier concentration. It is known that, in such a case of doping, an additional carrier scattering mechanism (i.e., alloy scattering) comes into play due to the random distribution of different atoms in the same lattice site [40,41]. This explains the reason for modest mobility in these samples.

Figure 6b shows the temperature dependent Seebeck coefficient (S) results. The Seebeck co-efficient being positive for all of the compositions over the entire temperature range indicates *p*-type charge carriers, which is in good agreement with the Hall measurement results. Interestingly, room temperature S-values marginally increase with dopant level and does not follow the expected trend according to the variation of carrier densities. However, such an anomalous change is difficult to explain. For samples doped with Bi, the S-value increases from ~60 µV/K at RT to ~90 µV/K at 523 K, yet these S-values are nowhere close to the state of the art *p*-type thermoelectric materials [9,15,42,43,44,45]. Though these experiments to improve the thermoelectric properties by highly crystallizing the glass compositions vastly improves σ values, S-values were drastically reduced because of systematic loss of characteristic telluride glass features, as telluride glasses are known for their exceptionally high Seebeck coefficient values [25,26,28].

For comparison, it is useful to mention the properties of undoped Ge-Te glass. At room temperature, it possess a high Seebeck coefficient of ~960 µV/K, but the electrical conductivity is too low (~10^−3^ S/m) [27,28,29].

The room-temperature electrical transport properties of some of the phases are presented in Table 3. This gives a general idea on the role of contribution of constituent phases to the properties. For example, XRD results in Figure 2 show more intense Te peaks for GTS-Bi05 and GTS-Bi10, while the Te peaks are less intense for GTS-Bi15. Moreover, GTS-Bi15 has proportionately more Bi-Ge-Te phases, which are far superior in conductivity (σ > 10^4^ S/m) when compared to the Te phase (σ ≈ 70 S/m). This reflects in the decreased values of σ for GTS-Bi05 and GTS-Bi10 and relatively higher σ for GTS-Bi15 (Figure 6a). As the physical properties for some of the phases are not yet known, a more cogent explanation could not be presented at this juncture.

The temperature dependence of the thermoelectric power factor, calculated using the electrical conductivity and Seebeck coefficient as S^2^σ, is displayed in Figure 6c. GTS-Cu15 and GTS-Bi15 have almost the same power factor values, and comparatively higher than the other samples. The power factor for these heavily-doped samples does not improve much with temperature. Once again, although these systems demonstrate decent levels of electrical conductivity, the mediocre Seebeck coefficient values in all cases reduces the power factor, which is almost one order of magnitude lower than the existing well-known p-type thermoelectric materials [9,17,49,50].

**Figure 7:**
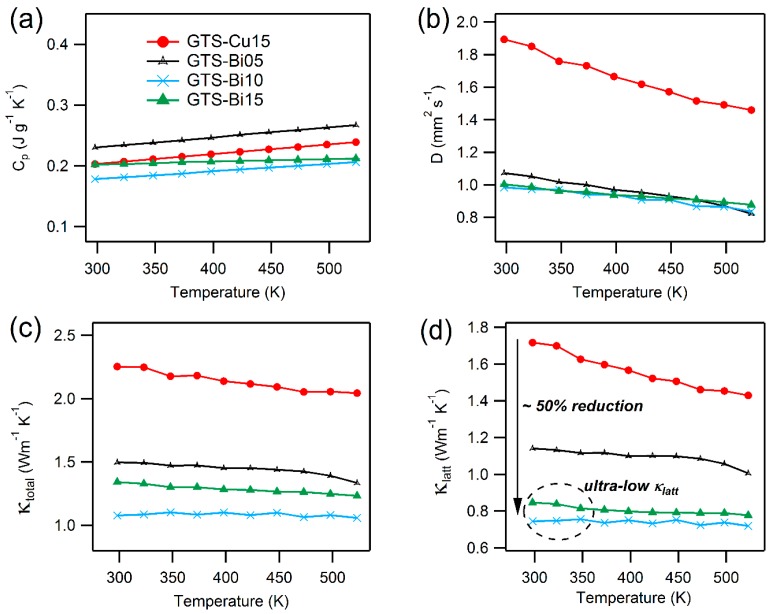
Temperature-dependent (**a**) specific heat capacity, *C_p_*; (**b**) thermal diffusivity, *D*; (**c**) total thermal conductivity, $\kappa_{total}$; and (**d**) lattice thermal conductivity, $\kappa_{latt}$ for Cu- and Bi-doped GTS samples. Color code legend applies to all of the plots.

Figure 7a,b displays the specific heat, *C_p_*, and thermal diffusivity, *D*, as a function of temperature. The measured *C_p_* values, within the experimental limits, are close to the values expected from Dulong-Petit law, represented in Equation (5):

(5) $$C_{p}=3R/M$$

where *R* is the gas constant and *M* is the molar mass. The temperature dependent total thermal conductivity, $\kappa_{total}$ derived from *D* and *C_p_* using Equation (4) is presented in Figure 7c. The lattice thermal conductivity ($\kappa_{latt}$) was estimated from $\kappa_{total}$ by subtracting the electronic contribution ($\kappa_{e}$) via the Wiedmann-Franz law, as in Equation (6), is shown in Figure 7d:

(6) $$\kappa_{e}=L\sigma T$$

where $\kappa_{e}$ is the electronic thermal conductivity and *L* is the Lorenz number computed by the condensed version of single parabolic band model with acoustic phonon scattering (SPB-APS), as in Equation (7) [51,52]:

(7) $$L=1.5+\exp\left[-\frac{\left|S\right|}{116}\right]$$

where the Seebeck coefficient (*S*) is in µV K^−1^ and Lorenz number (*L*) is in 10^−8^ WΩK^−2^. Temperature-dependent calculations for *L* and $\kappa_{e}$ for Cu and Bi doped GTS materials can be found in the Supporting Information (Figures S5 and S6).

As seen from the Figure 7c,d, the majority of the contribution for thermal conductivity comes from the lattice part. $\kappa_{total}$ for Bi-doped samples are relatively lower compared to the Cu-doped ones, due to the more metallic properties of Cu. Even though GTS-Cu15 and GTS-Bi15 possess almost the same power factor values and $\kappa_{e}$ values, GTS-Cu15 exhibits a $\kappa_{total}$ value of ~2.25 Wm^−1^ K^−1^ at room temperature, whereas GTS-Bi10 and GTS-Bi15 exhibit a $\kappa_{total}$ value of ~1.07 Wm^−1^ K^−1^ and ~1.3 Wm^−1^ K^−1^ at room temperatures, which is about a 50% reduction in comparison to that of the Cu-doped sample. This reduction is primarily because of significantly lower lattice contribution, presumably arising due to nanoprecipitate formation, which would produce effective phonon scattering in the lattices of heavily Bi-doped GTS samples. It has already been reported that Bi substitution in GeTe solid state solutions can result in segregation of Bi-rich nanoprecipitates [39]. In addition, such types of inclusions can cause collective phonon scattering from nanoprecipitates, meso-structured grain boundaries, and other crystallographic defects that could pave the way for reduction in lattice thermal conductivity [7,49,52]. In this work, for heavily-doped GTS-Bi samples, an ultra-low lattice thermal conductivity of ~0.7 Wm^−1^ K^−1^ was achieved at 523 K. $\kappa_{total}$ obtained for these doped crystalline materials; especially, the Bi-doped ones are essentially in the range with the $\kappa_{total}$ values of some of the well-known effective thermoelectric materials [15,42,49,53,54,55,56,57].

**Figure 8:**
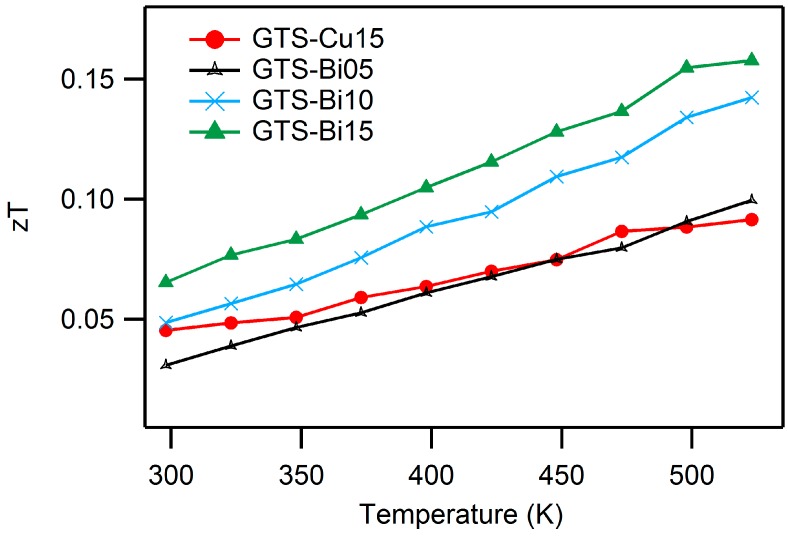
Dimensionless Figure of merit, *zT* for Cu- and Bi-doped GTS samples, showing a maximum *zT* of ~0.16 for GTS-Bi15 and ~0.092 for GTS-Cu15 at 523 K.

Though these heavily-doped GTS samples possess extremely low thermal conductivity, their *zT* values are quite low (Figure 8). It is their adversely low power factor that affects the overall *zT* of these materials, proving time and again that optimizing one parameter alone does not necessarily lead to improved efficiency, and an optimized blend of all properties is the indispensable criteria for an impactful thermoelectric device.

## 4. Conclusions

High-quality ingots of (Ge_20_Te_77_Se_3_)_100−*x*_M*_x_* (M = Cu or Bi; *x* = 5, 10, 15) were obtained using a vacuum sealed-tube melt quenching technique. With excess doping of Cu and Bi, the glassy network in pristine Ge_20_Te_77_Se_3_ was destroyed and highly-crystallized samples with multiple phases were produced. These p-type materials had high electrical conductivity (~8 × 10^4^ S/m) due to increased charge carrier density. Significantly lower total thermal conductivity was exhibited by these crystallized materials. Bi-doped samples demonstrated better thermoelectric features compared to Cu-doped samples. Moreover, TEM micrographs corroborated that heavily Cu-doped samples lack nano/meso-scale architectures. Ultra-low lattice thermal conductivity of ~0.7 Wm^−1^ K^−1^ was achieved for crystalline samples that were doped with 10 at % and 15 at % Bi, presumably due to Bi-rich nanoprecipitation. The high electrical conductivity coupled with low thermal transport provides the scope for further improvements in overall thermoelectric properties, especially the Seebeck coefficient, by proper optimization of parameters in crystallized glass compositions.

## Figures and Tables

**Table 1:** Sample compositions and their notations.

M	X	Sample	Representation
Cu	0	Ge_20_Te_77_Se_3_	GTS
	5	(Ge_20_Te_77_Se_3_)_95_Cu_5_	GTS-Cu05
	10	(Ge_20_Te_77_Se_3_)_90_Cu_10_	GTS-Cu10
	15	(Ge_20_Te_77_Se_3_)_85_Cu_15_	GTS-Cu15
Bi	5	(Ge_20_Te_77_Se_3_)_95_Bi_5_	GTS-Bi05
	10	(Ge_20_Te_77_Se_3_)_90_Bi_10_	GTS-Bi10
	15	(Ge_20_Te_77_Se_3_)_85_Bi_15_	GTS-Bi15

**Table 2:** Hall measurement results for carrier concentration and mobility.

Sample	Carrier Concentration *n* (cm^−3^)	Mobility, µ (cm^2^ V^−1^ s^−1^)
GTS-Cu15	2.81 × 10^20^	24.25
GTS-Bi05	1.09 × 10^20^	36.5
GTS-Bi10	2.38 × 10^20^	16.8
GTS-Bi15	2.39 × 10^20^	25.57

**Table 3:** Electrical transport properties of constituent phases (at ~300 K).

Phases	σ (S/m)	S (µV/K)	References
Bi_2_GeTe_4_	5 × 10^4^	92	[46]
Bi_2_Ge_3_Te_6_	6 × 10^4^	32	[46,47]
GeTe	8 × 10^5^	25	[33,48]
Te	70	250	This work

## Supplementary Materials

The following are available online at www.mdpi.com/1996-1944/10/4/328/s1. Figure S1: DSC curves for glassy samples, Figure S2: PXRD of amorphous glass compositions, Table S1: Rietveld refinement parameters for GTS-Cu15, Figure S3: STEM chemical maps, Figure S4: Stacking faults in Cu_7−*x*_Te_4_, Figure S5: Lorenz number calculation, Figure S6: *κ_e_* derivation.
