# Supplementary material for: Thermoelectric Properties of Highly-Crystallized Ge-Te-Se Glasses Doped with Cu/Bi
Source: Materials (Basel). 2017 Mar 23;10(4):328. doi: 10.3390/ma10040328 (PMC5506923; doi:10.3390/ma10040328)
Supplement: Supplementary file 1 [file materials-10-00328-s001.pdf]

Thermoelectric Properties of Highly-Crystallized Ge–Te–Se Glasses Doped with Cu/Bi

Bhuvanesh Srinivasan [1], Catherine Boussard-Pledel [1], Vincent Dorcet [2], Manisha Samanta [3], Kanishka Biswas [3], Robin Lefèvre [4], Franck Gascoin [4], François Cheviré [1], Sylvain Tricot [5], Michael Reece [6] and Bruno Bureau [1,*]

[1] Équipe Verres et Céramiques, ISCR CNRS UMR 6226, Université de Rennes 1, Rennes 35042, France; bhuvanesh.srinivasan@univ-rennes1.fr (B.S.); catherine.boussard@univ-rennes1.fr (C.B.-P.); francois.chevire@univ-rennes1.fr (F.C.)
[2] PRATS, ISCR CNRS UMR 6226, Université de Rennes 1, Rennes 35042, France; vincent.dorcet@univ-rennes1.fr
[3] New Chemistry Unit, Jawaharlal Nehru Centre for Advanced Scientific Research, Bangalore 560064, India; manishas@jncasr.ac.in (M.S.); kanishka@jncasr.ac.in (K.B.)
[4] Normandie Université, ENSICAEN, UNICAEN, CNRS, IUT-Caen, CRISMAT, 14050 Caen, France; robin.lefevre@ensicaen.fr (R.L.); franck.gascoin@ensicaen.fr (F.G.)
[5] Institut de Physique de Rennes, CNRS UMR 6251-Université de Rennes 1, Rennes 35042, France; sylvain.tricot@univ-rennes1.fr
[6] School of Engineering and Materials Science, Queen Mary University of London, London E1 4NS, UK; m.j.reece@qmul.ac.uk
* Correspondence: bruno.bureau@univ-rennes1.fr; Tel.: +33-223-236-573; Fax: +33-223-235-611

Academic Editor: Paz Vaqueiro
Received: 10 January 2017; Accepted: 20 March 2017; Published: date

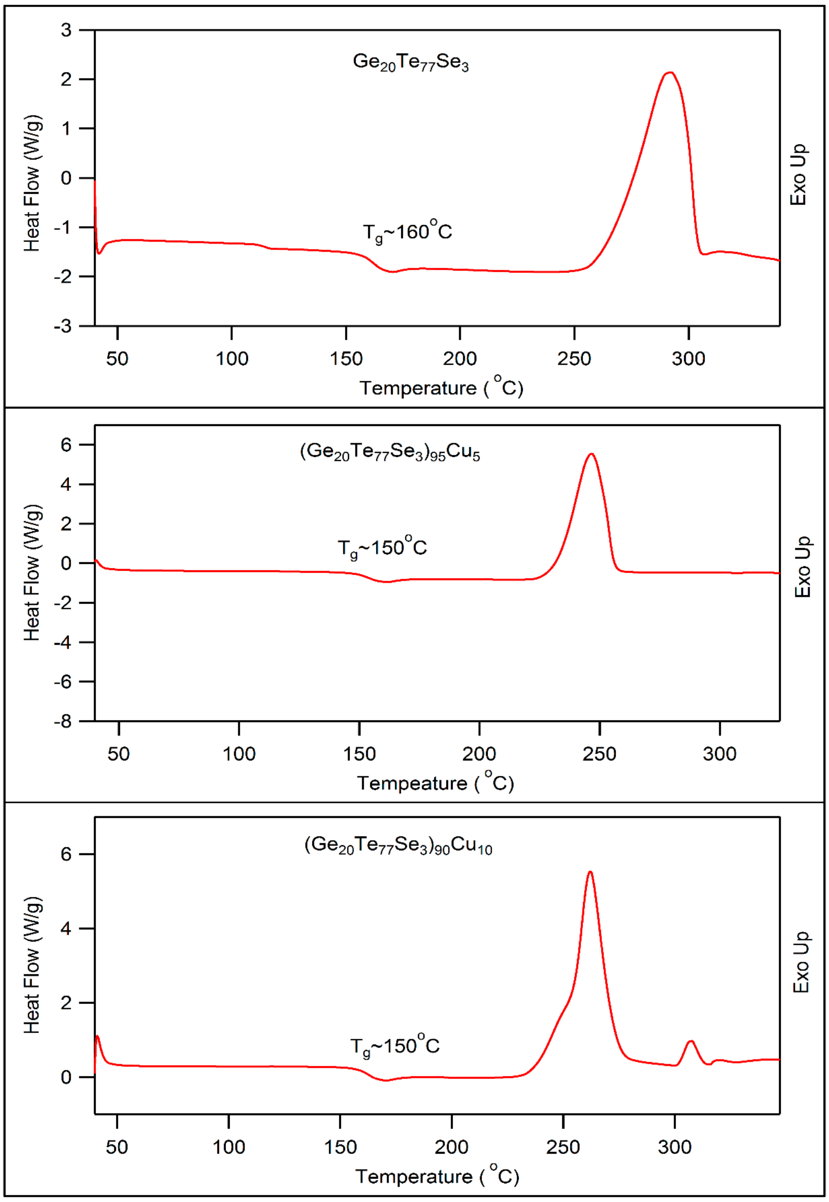

Figure S1. DSC curves for GTS, GTS-Cu05, and GTS-Cu10 samples. Comparing crystallization (T_c) and glass transition temperatures (T_g), it corresponds to $\Delta T \simeq 100$ °C resulting in quite stable glasses.

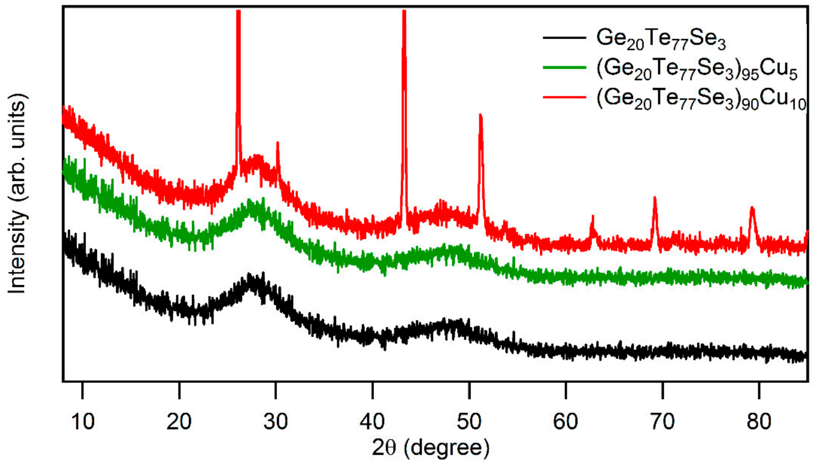

Figure S2. PXRD patterns showing completely amorphous features for GTS and GTS-Cu05, and with a few crystalline peaks for mostly amorphous GTS-Cu10.

Table S1. Details of the Rietveld refinement and quantitative analysis results for GTS-Cu15.

Phase	Cu_2GeTe_3	Te	GeTe
		**Lattice**	
a (Å)	5.9188(9)	4.4626(7)	4.1714(9)
b (Å)	5.9188(9)	4.4626(7)	4.1714(9)
c (Å)	5.9188(9)	5.9194(10)	10.621(3)
Space group	Cubic $F\bar{4}3m$	Trigonal $P3_121$	Rhombohedral $R3m$
		**Refinement**	
Nb. Background points		19	
Nb. Refined parameters		48	
Nb. Wyckoff positions	2	1	1
Nb. atoms	3	1	2
R_{Bragg}	3.39	2.99	3.54
Rf-factor	2.23	1.77	2.48
R_p		13.0	
R_{wp}		12.2	
R_{exp}		8.74	
Chi2		1.94	
		**Quantitative analysis**	
Weight content (%)	55.6 (0.8)	29.7 (0.5)	14.7 (0.6)

It is worth mentioning that, both in Cu- and Bi-doped samples, high resolution EDX predicted the presence of very few Se (< 1.5 at%), indicating that it could have crystallized into the main phase. Considering its negligible proportion and closely similar diffraction peak positions with Te, it is not taken into account for Rietveld refinement.

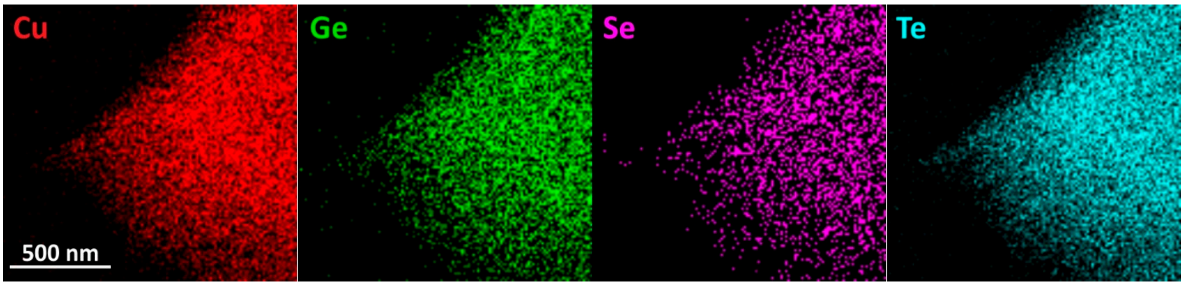

Figure S3. STEM micrographs of GTS-Cu15 showing chemical mapping images, demonstrating homogeneous distribution of elements in the post-synthesized ingots.

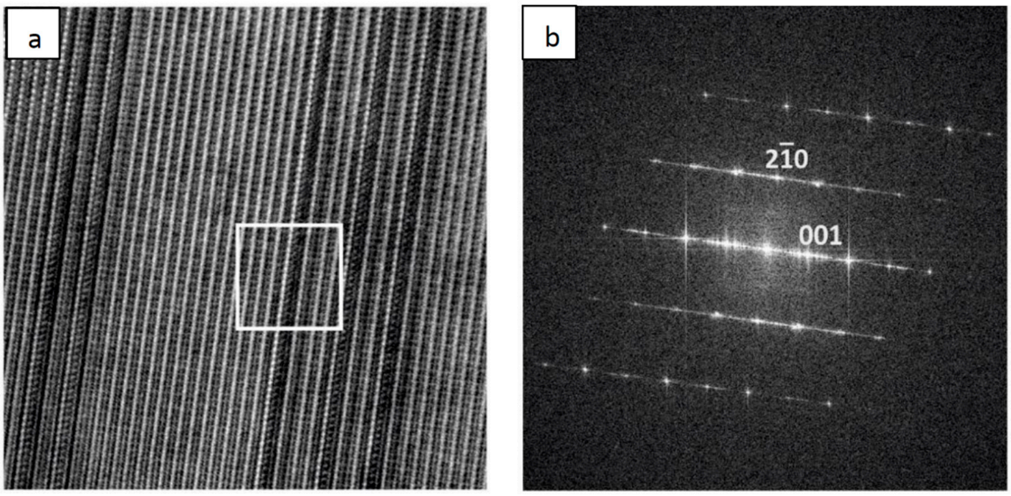

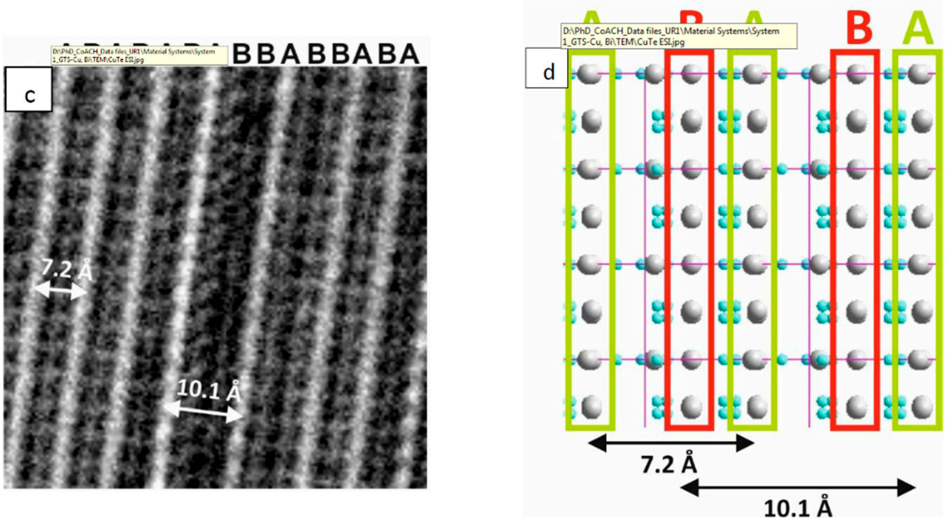

Figure S4. (**a**) HRTEM image of ion-beam modified region in GTS-Cu15 showing stacking faults in the $Cu_{7-x}Te_4$ phase (EDS Cu/Te = 1.16) oriented along the [120] zone axis, as shown in the fast Fourier Transform of the image in (**b**). (**c**) On enlarging the image, stacking fault along the c-axis with two different spacing between “white” planes is apparent: 7.2 Å and 10.1 Å, are seen. The investigation of the structure of Cu_7Te_4 [Baranova, R.V. Kristallografiya (1967) 12, (2) p. 266–273] along the zone axis [120] points to two kind of “Te” planes (A and B on the figure) with different spacing between them, as in figure (**d**). The 7.2Å spacing correspond to the c unit cell parameter of Cu_7Te_4, whereas the 10.1 Å spacing corresponds to the c-parameter plus the shortest distance between A and B, which means that we have twice as many examples of the shortest distances than the longest distances in these areas.

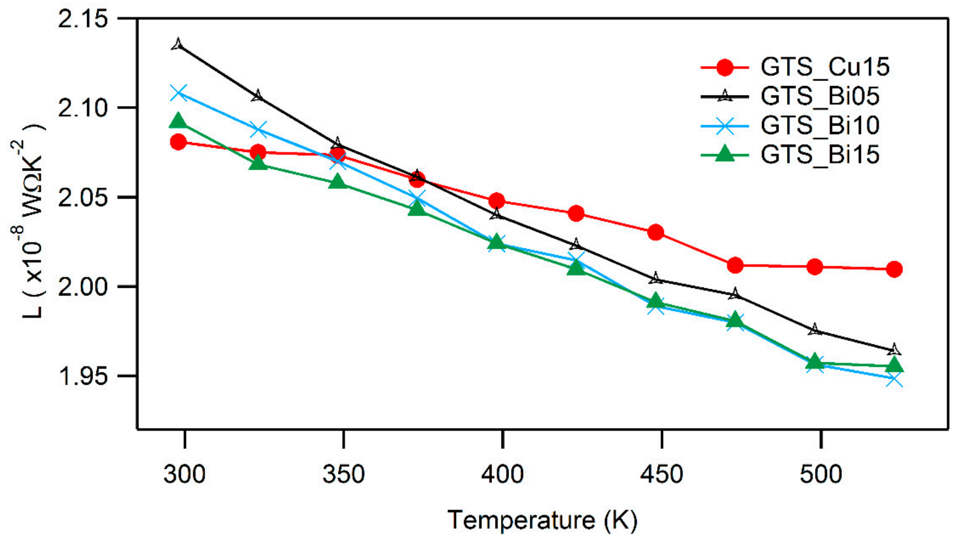

Figure S5. Temperature-dependent Lorenz number, L computed by the condensed version of single parabolic band model with acoustic phonon scattering (SPB-APS):

$$L = 1.5 + \exp\left[-\frac{|S|}{116}\right]$$

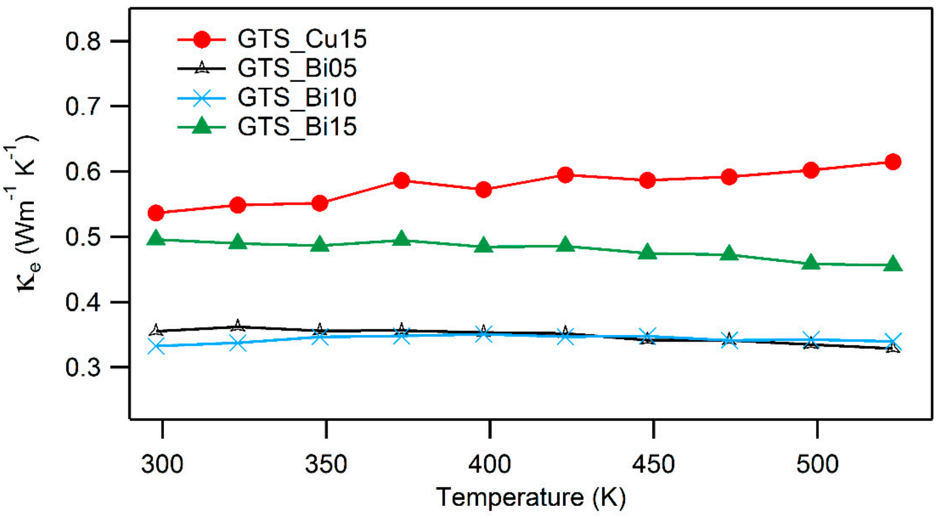

Figure S6. Temperature-dependent electronic thermal conductivity, κe calculated from Wiedmann-Franz law:

$$\kappa_e = L\sigma T$$
